# A Case of Concomitant Lung Adenocarcinoma and Pleural Metastasis of Papillary Thyroid Carcinoma With BRAF V600E Mutation

**DOI:** 10.1002/rcr2.70119

**Published:** 2025-02-13

**Authors:** Akinari Atsumi, Tetsuo Tani, Kota Ishioka, Keisuke Nishikawa, Yasuhide Okamoto, Saeko Takahashi

**Affiliations:** ^1^ Division of Pulmonary Medicine Saiseikai Central Hospital Tokyo Japan; ^2^ Department of Otorhinolaryngology Saiseikai Central Hospital Tokyo Japan

**Keywords:** BRAF V600E mutation, concomitant cancer, non‐small cell lung carcinoma, papillary thyroid carcinoma

## Abstract

A 62‐year‐old woman with a history of papillary thyroid carcinoma presented to our hospital with fever and cough and was diagnosed with stage IV non‐small cell lung carcinoma (NSCLC). One year after chemoimmunotherapy, a re‐biopsy of the left pleural tumour lesion was performed. Histological analysis revealed papillary thyroid carcinoma. Another biopsy was performed on the primary tumour, and the histological analysis of the primary tumour lesion confirmed NSCLC. BRAF V600E mutations were detected in both left pleural metastatic lesions of papillary thyroid carcinoma and the primary tumour of NSCLC. Dabrafenib and trametinib reduced both tumour lesions. Here, we report a rare case of concomitant BRAF V600E‐mutated NSCLC and pleural metastasis from papillary thyroid carcinoma.

## Graphical abstract

Recent molecular characterizations of multiple cancers have enabled the identification of driver oncogenes, and multiple molecular‐targeted inhibitors have been developed. We report the first case, to our knowledge, of concomitant lung carcinoma and pleural metastasis of papillary thyroid carcinoma with BRAF V600E mutation.

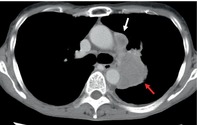

## 1 Introduction

Recent molecular characterizations of multiple cancers have enabled the identification of driver oncogenes, and multiple molecular‐targeted inhibitors have been developed. BRAF mutations have been identified in approximately 5% of all malignancies, with the highest incidence observed in melanoma (39.7%), thyroid cancer (33.3%), small intestinal malignancies (8.9%), and non‐small cell lung cancer (NSCLC) (2%–5%) [1]. The BRAF V600E mutation is the most common BRAF mutation; it plays a role as an oncogenic driver gene and is a target for molecular targeted therapy.

Concomitant BRAF V600E‐positive cancers, such as lung cancer and hairy cell leukaemia, or malignant melanoma and hairy cell leukaemia, have been reported [2, 3]. However, there have been no reports of concomitant non‐small‐cell carcinoma and thyroid carcinoma. We report the first case of concomitant lung carcinoma and pleural metastasis of papillary thyroid carcinoma with BRAF V600E mutation.

## 2 Case Report

**FIGURE 1:**
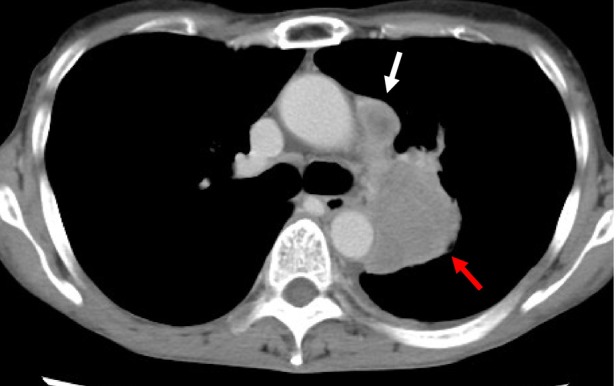
Computed tomography (CT) showing a mass in the left lung (red arrow), lymph node swelling (white arrow), and small amount of pleural effusion (A).

**FIGURE 2:**
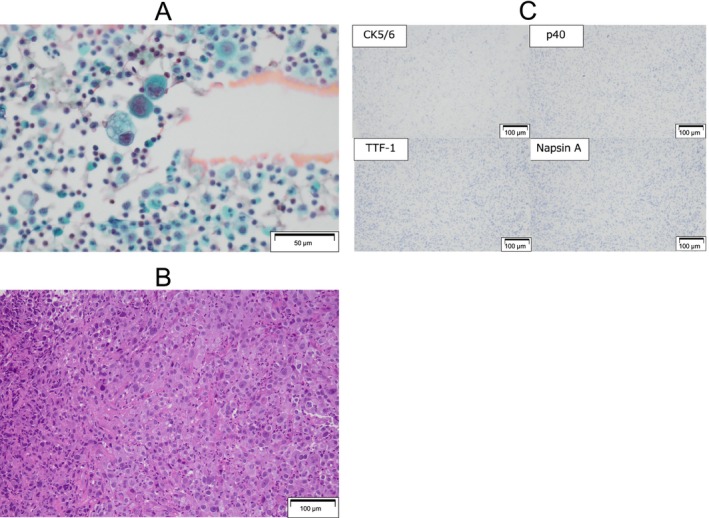
Cytological examination of pleural effusion (A). Haematoxylin and eosin staining (B) and immunohistochemical staining (C) of lung biopsy specimens via bronchoscopy.

A 62‐year‐old female had a history of right lobe thyroidectomy for papillary thyroid carcinoma with lymph node metastasis at age 20. She has had no recurrence or family history of lung or thyroid carcinoma. One year before the admission, the patient presented to our hospital exhibiting symptoms of fever and cough, and radiographic imaging examination via computed tomography (CT) revealed a mass in the left lung and lymph node swelling, and small amount of left malignant pleural effusion (Figure 1) which was confirmed cytology (Figure 2A). Subsequent bronchoscopy examination confirmed the diagnosis of NSCLC histologically (Figure 2B), and the results were consistent with the prior cytological findings. The tumour was negative for TTF‐1, napsin A, CK5/6, and p40 staining by IHC (Figure 2C) and was diagnosed as NOS (not otherwise specified). Genetic panel testing was not performed because of the insufficient tumour volume.

**FIGURE 3:**
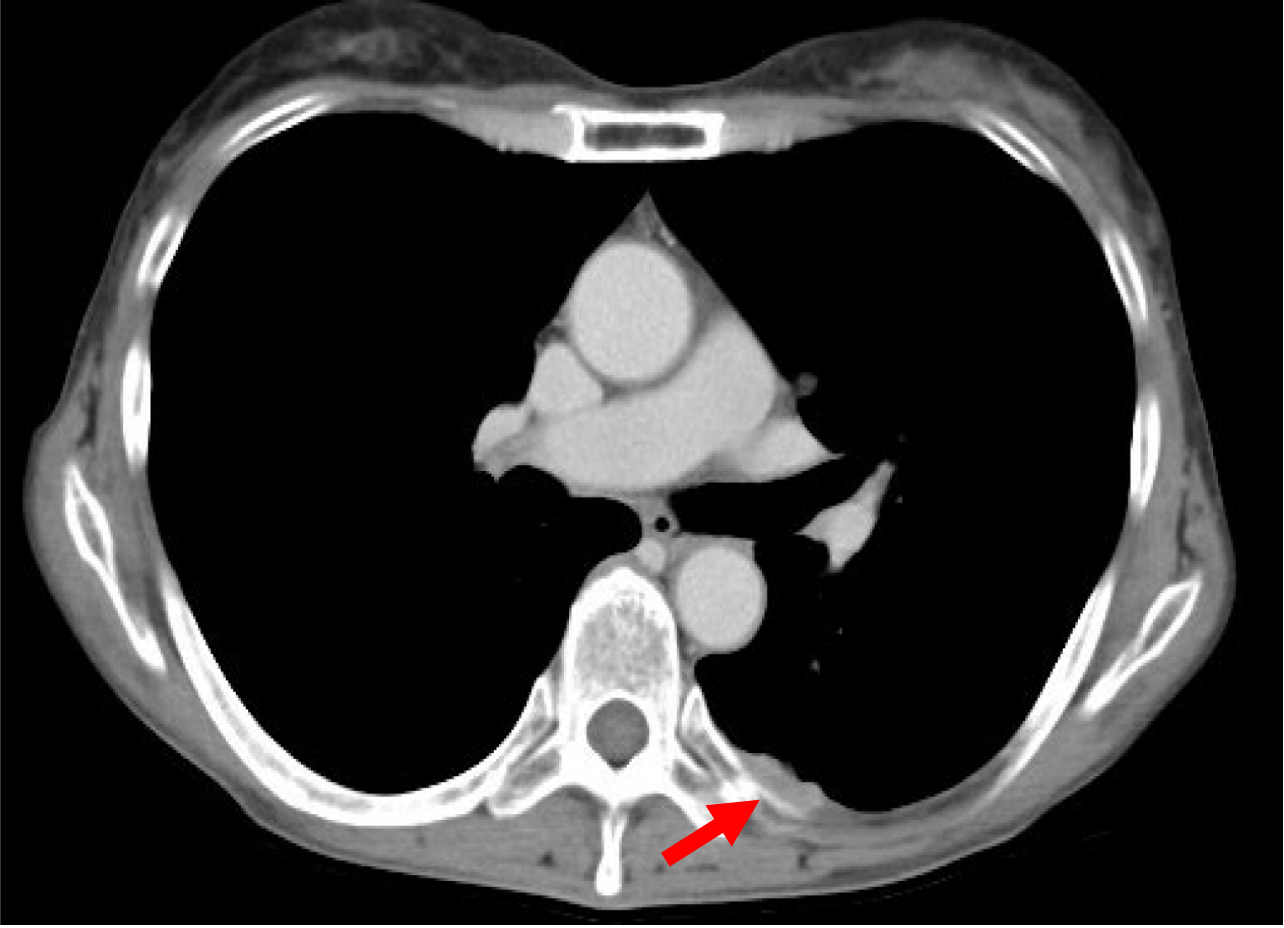
CT tomography shows a pleural nodule (red arrow).

**FIGURE 4:**
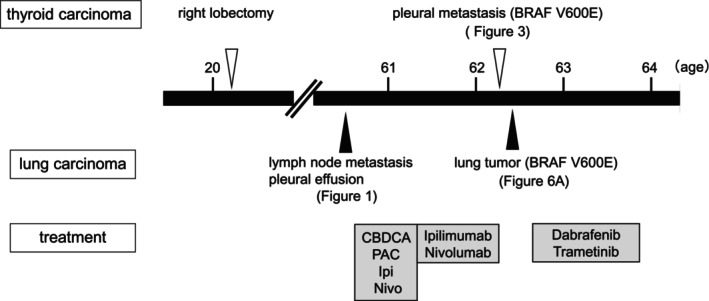
Timeline summarising the clinical course of this case.

The patient was diagnosed with stage IVA NSCLC and was treated with carboplatin, paclitaxel, ipilimumab, and nivolumab for two cycles, followed by maintenance therapy with ipilimumab and nivolumab. Fourteen months post‐treatment, a computed tomography (CT) scan identified a slight enlargement of the primary lesion and the significant progression of pleural nodule (Figure 3). Therefore, a thoracoscopic biopsy of the left pleural lesion was performed to evaluate the histopathological examination and genetic mutations (Figure 4).

**FIGURE 5:**
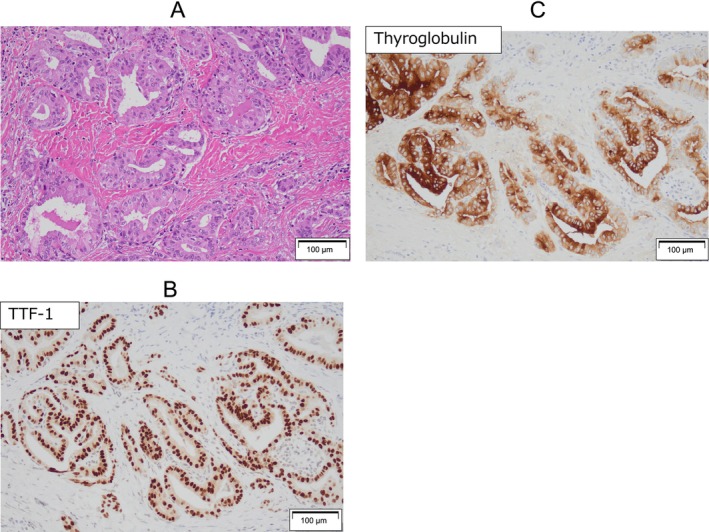
Pathological examination of the lung biopsy specimens. Microscopic examination revealed an adenocarcinoma. Thyroid‐follicle formation (A). Immunohistochemistry (B and C).

Histological findings of the left pleural mass showed adenocarcinoma, thyroid follicle formation (Figure 5A), and immunohistochemistry demonstrated strong and diffuse positive staining for TTF‐1 (Figure 5B) and thyroglobulin (Figure 5C) in over 90% of tumour cells, leading to the diagnosis of pleural metastasis from papillary thyroid carcinoma.

Subsequently, bronchoscopic biopsy of the primary lung mass was performed. Histological findings of the re‐biopsy samples were consistent with those obtained at the time of lung carcinoma diagnosis via IHC staining. Mutation analysis of the primary lung tumour detected a BRAF V600E mutation using AmoyDx. The additional mutation analysis of the left pleural metastatic lesion of papillary thyroid carcinoma also detected BRAF V600E mutation by Oncomine DxTT. Therefore, both lung and thyroid carcinomas were concomitantly diagnosed as BRAF V600E‐positive cancers. Radioactive iodine ablation was considered following total thyroidectomy for thyroid carcinoma. However, since lung carcinoma was identified as the prognostic factor, treatment was selected accordingly.

**FIGURE 6:**
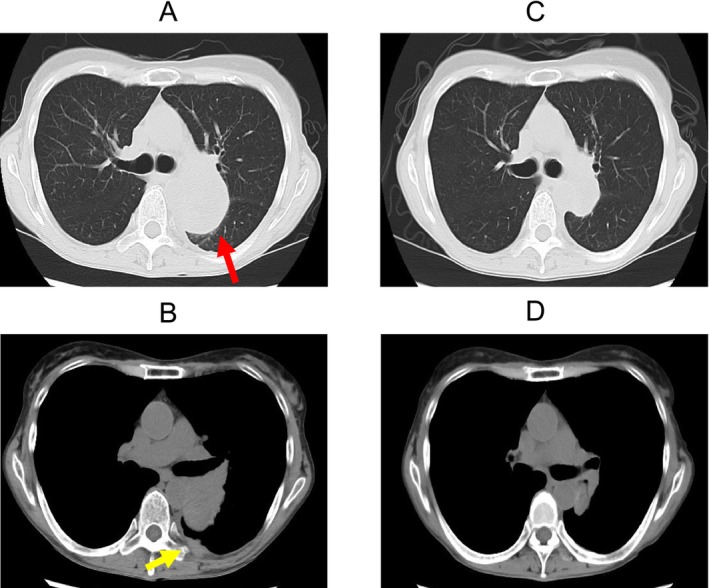
CT images showing a 63 × 42 mm tumour in the left lung (red arrow, A) and pleural metastasis (yellow arrow, B) prior to treatment. After 52 days of treatment, the tumour size reduced to 33 × 24 mm (C), and no pleural nodules were observed (D).

The patient received dabrafenib (150 mg orally twice daily) and trametinib (2 mg orally daily), which reduced both the primary lung tumour and the left pleural metastatic lesion volume. CT images showed a 63 × 42 mm tumour in the left lung (Figure 6A) and pleural metastasis (Figure 6B) prior to treatment. After 52 days of treatment, the tumour size reduced to 33 × 24 mm (Figure 6C), and no pleural nodules were observed (Figure 6D).

There have been no signs of relapse after the administration of dabrafenib and trametinib for 12 months.

## 3 Discussion

This report describes a rare case of concomitant BRAF V600E‐positive NSCLC and pleural metastasis of papillary thyroid carcinoma. While BRAF V600E mutations have been observed in multiple types of cancers, such as lung carcinoma and haematological malignancies like hairy cell leukaemia, there is no reports of the simultaneous occurrence of BRAF V600E mutations in both NSCLC and papillary thyroid carcinoma with pleural metastasis. This case emphasises the importance of molecular profiling and the critical role of re‐biopsy in guiding treatment, particularly when multiple tumour types are present.

BRAF mutations are detected across a broad range of cancers and are categorised into three functional classes. The BRAF V600E mutation, which is the most common point mutation among BRAF mutations in all histological types, is a class I BRAF mutation that has high kinase activity and results in constitutive activation of the MAPK signalling pathway, driving tumour initiation and progression across various malignancies [1]. In contrast, non‐BRAF V600E mutations, classified as class II or III, exhibit distinct biological and clinical behaviours. These mutations often have different downstream signalling effects and clinical implications compared to BRAF V600E mutations [1].

BRAF mutations are typically somatic mutations, but germline mutations can cause congenital syndromes like cardio‐facio‐cutaneous (CFC), Noonan, and Leopard syndromes [4, 5]. Most germline BRAF mutations involve non‐BRAF V600E mutations, whereas germline mutations in BRAF V600 are exceedingly rare, with only a single reported case [6]. Notably, the case is associated with CFC syndrome.

Therefore, BRAF germline mutations are compatible with human growth and development but were not observed in this case. The BRAF V600E mutation, in this case, is considered a somatic mutation, although both NSCLC and papillary thyroid carcinoma tumours have the BRAF V600E mutation.

Although concomitant BRAF V600E‐positive cancers, such as lung cancer and hairy cell leukaemia, or malignant melanoma and hairy cell leukaemia, have been reported [2, 3], Concomitant cancers are extremely rare and may provide important insights into the oncogenic mechanisms involving BRAF V600E mutations. Mutations in genes such as BRAF and KRAS are found in multiple types of malignancies. Therefore, the investigation of genetic mutations has the potential to facilitate molecularly targeted therapies.

In this case, the combination therapy of dabrafenib and trametinib, targeting BRAF V600E mutation, was highly effective. Both tumours showed significant shrinkage and sustained remission for 12 months, demonstrating the potential of BRAF‐targeted therapies for treating multiple BRAF V600E‐positive cancers. Dabrafenib and trametinib combination therapy is commonly used as the first‐line treatment for stage IV BRAF V600E mutation‐positive NSCLC. This therapeutic approach demonstrated a response rate of 64% and a reported progression‐free survival of 10.9 months [7]. Dabrafenib and trametinib combination therapy has also been approved for unresectable or metastatic solid tumours with BRAF V600E mutation [8]. Especially, radioiodine‐refractory thyroid carcinomas with BRAF V600E mutation, yielding a response rate of 48% and a progression‐free survival of 15.1 months [9]. It should be noted that although dabrafenib (150 mg orally twice daily) and trametinib (2 mg orally daily) have been used for BRAF V600E‐positive tumours in these trials, the doses in concomitant cases are still unknown.

This case underscores the presence of both carcinomas in the thoracic cavity, and gene analysis was performed using tumour species from the pleural metastasis and lung tumours. This suggests the potential for contamination of each cancerous lesion. However, this possibility is refuted by the treatment response of both pleural metastasis of papillary thyroid carcinoma and lung tumours of NSCLC to dabrafenib and trametinib combination therapy targeting BRAF V600E mutated tumour.

Genetic mutation testing for multiple tumours is routinely performed in clinical practice. However, a distinguishing feature of the present case was the simultaneous presence of the primary lung cancer and pulmonary metastasis from the thyroid cancer within the thoracic cavity. This case highlights the importance of a re‐biopsy.

In summary, this rare case of concomitant BRAF V600E‐mutated NSCLC and pleural metastasis of papillary thyroid carcinoma demonstrates the effectiveness of targeted therapy with dabrafenib and trametinib. The combination therapy resulted in sustained remission, underscoring the potential of BRAF‐targeted therapies in treating multiple BRAF V600E‐positive malignancies. This case also highlights the importance of molecular profiling and re‐biopsy in managing complex cancer cases, offering valuable guidance for future therapeutic strategies in similar situations.

## Author Contributions

Akinari Atsumi, Tetsuo Tani, and Saeko Takahashi wrote the manuscript. Akinari Atsumi, Tetsuo Tani, Kota Ishioka, Keisuke Nishikawa, Yasuhide Okamoto, and Saeko Takahashi were involved in intensive care management and review of the work.

## Ethics Statement

The authors declare that appropriate written informed consent was obtained for the publication of this manuscript and accompanying images.

## Conflicts of Interest

The authors declare no conflicts of interest.

## Data Availability

Data available on request from the authors.
